# The destiny of Ca^2+^ released by mitochondria

**DOI:** 10.1007/s12576-014-0326-7

**Published:** 2014-07-04

**Authors:** Ayako Takeuchi, Bongju Kim, Satoshi Matsuoka

**Affiliations:** 1 Department of Integrative and Systems Physiology, Faculty of Medical Sciences, University of Fukui, 23-3, Matsuokashimoaizuki, Eiheiji-cho, Yoshida-gun, Fukui, 910-1193 Japan; 2 Division of Experimental Immunology, Institute for Genome Research, University of Tokushima, 3-18-15, Kuramoto, Tokushima, 770-8503 Japan

**Keywords:** Mitochondria, Ca^2+^ dynamics, NCLX, Letm1, Cellular function

## Abstract

Mitochondrial Ca^2+^ is known to regulate diverse cellular functions, for example energy production and cell death, by modulating mitochondrial dehydrogenases, inducing production of reactive oxygen species, and opening mitochondrial permeability transition pores. In addition to the action of Ca^2+^ within mitochondria, Ca^2+^ released from mitochondria is also important in a variety of cellular functions. In the last 5 years, the molecules responsible for mitochondrial Ca^2+^ dynamics have been identified: a mitochondrial Ca^2+^ uniporter (MCU), a mitochondrial Na^+^–Ca^2+^ exchanger (NCLX), and a candidate for a mitochondrial H^+^–Ca^2+^ exchanger (Letm1). In this review, we focus on the mitochondrial Ca^2+^ release system, and discuss its physiological and pathophysiological significance. Accumulating evidence suggests that the mitochondrial Ca^2+^ release system is not only crucial in maintaining mitochondrial Ca^2+^ homeostasis but also participates in the Ca^2+^ crosstalk between mitochondria and the plasma membrane and between mitochondria and the endoplasmic/sarcoplasmic reticulum.

## Introduction

 Mitochondria are crucial organelles in ATP production as well as in Ca^2+^ storage. They also serve as master switches determining cell fate on exposure to different stimuli [1–3]. Mechanisms of Ca^2+^ homeostasis in mitochondria have been extensively studied over the last half century, so the importance of mitochondrial Ca^2+^ in regulating mitochondrial functions is well recognized. Ca^2+^ enters mitochondria mainly via a mitochondrial Ca^2+^ uniporter, a protein known as MCU or CCDC109A [4, 5]. The characteristics and physiological and pathophysiological functions of this protein, and its associated proteins have been widely studied [6–8]. On the other hand, studies of the molecules responsible for Ca^2+^ release by mitochondria have just begun, although functional characterization of the release system started in the 1970s [9]. The mitochondrial Ca^2+^ release system mainly consists of an Na^+^–Ca^2+^ exchanger and an H^+^–Ca^2+^ exchanger. The molecule responsible for the former (NCLX) was identified in 2010 [10]. A possible molecular candidate for the latter (Letm1) was reported in 2009 [11], although this is still controversial.

It is now well understood that some mitochondria are in close contact with the plasma membrane and others with the endoplasmic reticulum (ER)/sarcoplasmic reticulum (SR). Although the molecular mechanisms of tethering of mitochondria to the plasma membrane are not well understood, several tethering protein complexes involved in interactions between mitochondria and the ER/SR have been identified, and details of the molecular mechanisms have been reviewed [12, 13]. It is believed that these interactions are important in modulating a variety of cellular functions.

In this paper we review recent progress in the study of mitochondrial Ca^2+^ release system, specifically, interactions between mitochondria and the ER/SR and interactions between mitochondria and the plasma membrane, and discuss their physiological and pathophysiological significance, focusing on the destiny of Ca^2+^ released by mitochondria.

## Physiological roles of Ca^2+^ in mitochondria and released by mitochondria

### Mitochondrial Ca^2+^ regulates diverse cellular functions

**Fig. 1:**
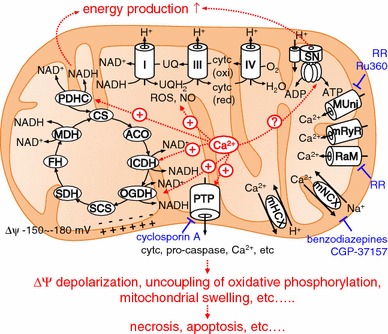
Regulation of mitochondrial functions by mitochondrial Ca^2+^. Mitochondrial Ca^2+^ activates three dehydrogenases in the mitochondrial matrix: pyruvate dehydrogenase (PDHC), oxoglutarate dehydrogenase (OGDH), and isocitrate dehydrogenase (ICDH). ROS production is stimulated by increased mitochondrial Ca^2+^, possibly via increased NADH production. F1-Fo ATP synthase is activated by mitochondrial Ca^2+^, although this is still controversial. This regulatory activity contributes to energy homeostasis. PTP opening is activated by a large increase of mitochondrial Ca^2+^, resulting in the release of a variety of compounds from mitochondria, for example cytochrome c (cytc), pro-caspase, and Ca^2+^, leading to apoptosis or necrosis. *CS* citrate synthase, *ACO* aconitase, *SCS* succinyl-CoA synthase, *SDH* succinate dehydrogenase, *FH* fumarate hydratase, *MDH* malate dehydrogenase, *SN* F1-Fo ATP synthase, *RR* ruthenium red, *MUni* mitochondrial Ca^2+^ uniporter, *mRyR* mitochondrial RyR, *mNCX* mitochondrial Na^+^–Ca^2+^ exchanger, *mHCX* mitochondrial H^+^–Ca^2+^ exchanger

It is now well accepted that mitochondrial Ca^2+^ is important in regulation of diverse cellular functions (Fig. 1). For example, an increase of mitochondrial Ca^2+^ activates three dehydrogenases in the mitochondrial matrix: pyruvate dehydrogenase, oxoglutarate dehydrogenase, and isocitrate dehydrogenase. As a result, the mitochondrial NADH-to-NAD ratio increases and, hence, flow of electrons down the respiratory chain increases, adjusting ATP synthesis to the increased ATP needs of a cell [14–16]. Mitochondrial Ca^2+^-mediated NADH oxidase activation may result in increased production of mitochondrial reactive oxygen species (ROS). In fact, an increase of mitochondrial Ca^2+^ has been reported to increase production of ROS in the heart and in neurons, resulting in impaired respiration and in cytotoxicity [17, 18]. Several studies have indicated that Ca^2+^ activates F1-Fo ATP synthase, balancing ATP utilization increased by Ca^2+^, and that it is probably the intra-mitochondrial Ca^2+^ which regulates F1-Fo ATP synthase, although this is still controversial [19, 20]. A large increase of mitochondrial Ca^2+^ results in opening of a non-specific pore called the mitochondrial permeability transition pore (PTP). PTP opening is followed by inner mitochondrial membrane depolarization, uncoupling of oxidative phosphorylation, and massive mitochondrial swelling, resulting in necrosis [1, 21–23]. PTP is also involved in apoptosis. Apoptosis-promoting factors, for example cytochrome c and pro-caspases, are released to the cytosol through PTP [24–26]. Furthermore, cardiac mitochondrial nitric oxide (NO) synthase is activated by mitochondrial Ca^2+^, contributing to NO-mediated cardioprotection against PTP opening [27]. Accordingly, mitochondrial Ca^2+^ is crucial in tuning a variety of mitochondrial and, thus, cellular functions.

**Fig. 2:**
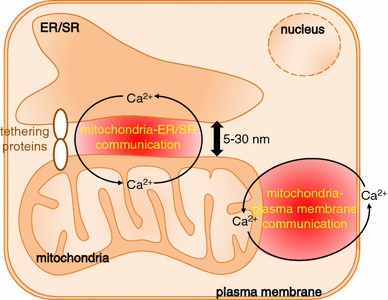
Ca^2+^ communication between mitochondria and the plasma membrane and between mitochondria and the ER/SR

What is the source of the mitochondrial Ca^2+^? The location of the mitochondria within the cell seems important for mitochondrial Ca^2+^ uptake. Some mitochondria are located in proximity to the plasma membrane or the ER/SR. Ca^2+^ flowing through the plasma membrane or released from the ER/SR predominantly enters neighbouring mitochondria. In other words, there is Ca^2+^ crosstalk between mitochondria and the plasma membrane and between mitochondria and the ER/SR (Fig. 2). A substantial amount of the Ca^2+^ flowing through the plasma membrane or released by the ER/SR binds to cytosolic soluble Ca^2+^-binding proteins, for example calmodulin, troponin C, and calbindin. Of these, calmodulin is a ubiquitous protein well conserved across eukaryotes and Ca^2+^-bound calmodulin regulates a variety of cellular functions by binding to calmodulin-dependent kinase, calcineurin, plasma membrane Ca^2+^ ATPase, and the ryanodine receptor, among others [28]. Further information about these Ca^2+^-buffering proteins is available in a detailed review [29].

### Ca^2+^ crosstalk between mitochondria and the plasma membrane and between mitochondria and the ER/SR

Ca^2+^ enters cells via Ca^2+^ channels in the plasma membrane, for example the voltage-gated Ca^2+^ channel (VDCC) and the Ca^2+^ release-activated Ca^2+^ channel (CRAC) or store-operated Ca^2+^ channel. The Ca^2+^ which enters the cell is efficiently taken up by mitochondria located near the plasma membrane [30–33]. Lawrie et al. [30] found that after depletion of stored Ca^2+^, re-addition of extracellular Ca^2+^ evoked an increase in mitochondrial Ca^2+^ but not in cytoplasmic Ca^2+^ in human umbilical vein endothelial cell line ECV304, in which 14 % of mitochondria are located within 700 nm of the inner surface of the plasma membrane. They suggested that Ca^2+^ levels increase in microdomains beneath the plasma membrane, causing predominant Ca^2+^ uptake by mitochondria facing the microdomains. However, the contribution of the Ca^2+^ microdomains depends on cell type, because this phenomenon was not observed for a clone of a HeLa cell line, in which <6 % of mitochondria are located in the proximity of the plasma membrane. Park et al. [32] reported that store-operated Ca^2+^ influx via CRAC channels through the basolateral membrane led to predominant Ca^2+^ uptake by sub-plasmalemmal mitochondria in pancreatic acinar cells. These reports suggest Ca^2+^ communication, through plasma membrane Ca^2+^ channels, with mitochondria located in proximity to plasma membrane. Ca^2+^ communication in the opposite direction has also been suggested. Mitochondrial membrane depolarization suppressed store-operated Ca^2+^ entry in T lymphocytes and in rat basophilic leukaemia cells [34, 35]. Accordingly, bidirectional Ca^2+^ crosstalk between mitochondria and the plasma membrane is important in the regulation of cellular functions. As will be described below, the mitochondrial Na^+^-Ca^2+^ exchanger NCLX is involved in this Ca^2+^ crosstalk.

**Fig. 3:**
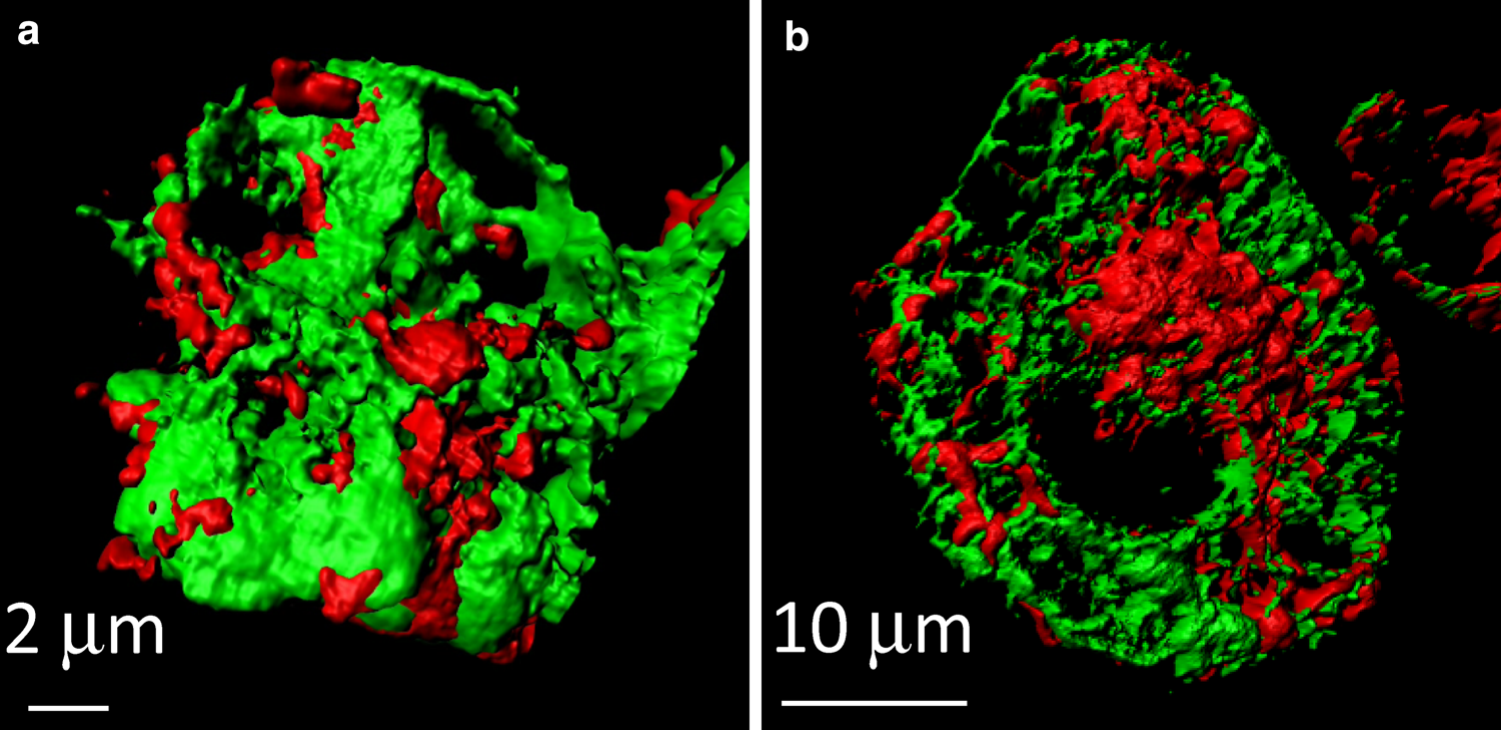
Close location of mitochondria and the ER/SR in A20 B lymphocytes (**a**) and in HL-1 cardomyocytes (**b**). Cells were co-transfected with mitochondria-targeted pTagRFP-mito (*red*) and ER/SR-targeted cameleon D1ER (*green*). Images were acquired by use of a laser-scanning confocal microscope (LSM710, Carl Zeiss) with a ×63 oil objective lens, and 3D images were reconstructed by use of Imaris (Bitplane) (color figure online)

The ER/SR is major Ca^2+^ store within the cells. The ER/SR is located close to mitochondria, approximately 5–30 nm, and narrow interorganellar spaces exist between the organelles in a variety of cell types, including B lymphocytes and cardiomyocytes [36]. Figure 3 shows 3D-reconstructed confocal images of mitochondria and the ER/SR of cultured B lymphocytes and cardiomyocytes. The two organelles are located close to each other, although narrow interorganellar spaces are not visible. The close contact between mitochondria and the ER/SR is supported by tethering proteins, for example MIRO, DRP1, MFN2, and Mmm1/Mdm10/Mdm12/Mdm34 complex [12, 13]. Mitochondria–ER/SR communication is important for regulation of a variety of cellular processes, including lipid biosynthesis, mitochondrial division, and Ca^2+^ signalling [12, 37]. With regard to Ca^2+^, these narrow interorganellar spaces may serve to create Ca^2+^ level much higher than in the bulk cytosol, up to 9–50 μM [38, 39], enabling the mitochondrial Ca^2+^ uniporter, the Ca^2+^ affinity of which is relatively low, to transport enough Ca^2+^ into mitochondria. Several studies reported that an inositol 1,4,5-trisphosphate (IP_3_) receptor of the ER and a voltage-dependent anion channel of the outer mitochondrial membrane are located in the mitochondria–ER region, creating a Ca^2+^ pathway from the ER to the mitochondria. This may aid regulation of energy metabolism and apoptosis in such cells as HeLa cells, CHO cells, fibroblasts, and yeast [40–42]. Ca^2+^ released from the ryanodine receptor (RyR) on the SR also accumulates in mitochondria of rat ventricular myocytes, indicating preferential coupling of Ca^2+^ transport from the SR to mitochondria [38]. It has been suggested that reverse Ca^2+^ movement, from mitochondria to the ER/SR, is important for refilling ER/SR Ca^2+^ after ligand stimulation of such cells as HeLa cells, endothelial cell line, and vascular smooth muscle cells, to minimize cytosolic Ca^2+^ elevation and to prevent depletion of ER/SR Ca^2+^ [43–45]. The crucial importance of NCLX in the movement of Ca^2+^ from mitochondria to the ER/SR was clearly demonstrated in our recent studies [46–48], as described below. The bidirectional Ca^2+^ crosstalk between mitochondria and the ER/SR is also important for regulating cellular functions.

Taken together, Ca^2+^ crosstalk between mitochondria and the plasma membrane and between mitochondria and the ER/SR is important in controlling cellular functions. Understanding the roles of each molecule participating in the Ca^2+^ crosstalk is particularly important for fully understanding cellular physiological and pathophysiological functions.

## Ca^2+^-transporting systems in mitochondria

Studies of mitochondrial Ca^2+^ dynamics started more than half a century ago. The existence of a respiration-dependent pathway of Ca^2+^ into isolated rat kidney mitochondria was reported in the early 1960s [49, 50]. In the 1970s, pathways of Ca^2+^ out of isolated rat heart mitochondria (Na^+^-dependent) and out of isolated rat liver mitochondria (Na^+^-independent and H^+^-dependent) were discovered [9, 51]. Since then, characteristics of mitochondrial Ca^2+^ dynamics have been extensively studied [1, 52–54].

The major pathways of Ca^2+^ uptake into and efflux out of mitochondria are summarized in Fig. 1. Ca^2+^ uptake into mitochondria is mainly mediated by a mitochondrial Ca^2+^ uniporter driven by a highly negative membrane potential (mitochondrial membrane potential Δ*Ψ* is −150 to −180 mV) [55]. Another uptake system is a rapid uptake mode (RaM) which might contribute to mitochondrial Ca^2+^ uptake from fast cytosolic Ca^2+^ transients. A mitochondrial ryanodine receptor is also reported to mediate Ca^2+^ uptake into rat heart mitochondria. Details are available in other reviews [1, 52]. It was not until the 2010s that the identities of the molecules responsible for the mitochondrial Ca^2+^ uniport were revealed. A regulator of the Ca^2+^ uniporter, MICU, was discovered in 2010 before cloning of the mitochondrial Ca^2+^ uniporter [56]. MCU (or CCDC109A) was then discovered as a gene coding the mitochondrial Ca^2+^ uniporter [4, 5]. Very recently, characteristics of MCU knockout have been reported. Although MCU knockout in *Trypanosoma brucei* resulted in marked dysregulation of mitochondrial bioenergetics, causing autophagy and cell death [57], relatively minor alteration of basal energetics was observed for MCU knockout mice [58]. MCU and its regulators have been reviewed in detail elsewhere [7, 8].

Ca^2+^ efflux from mitochondria is mainly mediated by two saturable pathways, an Na^+^-dependent (Na^+^–Ca^2+^ exchanger; benzodiazepines and CGP-37157-sensitive) pathway and an Na^+^-independent (H^+^–Ca^2+^ exchanger; ruthenium red-insensitive) pathway. Under pathophysiological conditions in which the PTP opens, PTP functions as a Ca^2+^ transporter from mitochondria. In the sections below we describe, in detail, the two physiological pathways of mitochondrial Ca^2+^ release.

### Mitochondrial Na^+^–Ca^2+^ exchanger

#### Biophysical properties of the mitochondrial Na^+^–Ca^2+^ exchanger

The mitochondrial Na^+^–Ca^2+^ exchanger was first discovered by Carafoli et al. [9] in 1974 in isolated rat heart mitochondria. This Na^+^–Ca^2+^ exchange activity is found in a wide variety of tissues and is dominant in the heart, brain, skeletal muscle, parotid gland, adrenal cortex, and brown fat [52, 59]. The Na^+^–Ca^2+^ exchanger is also present in liver, kidney, and lung mitochondria, although its activity is weak [60]. In tissues in which mitochondrial Na^+^–Ca^2+^ exchange activity is low, H^+^–Ca^2+^ exchange activity is of dominant importance in the release of Ca^2+^ from mitochondria [61].

One interesting characteristic of the mitochondrial Na^+^–Ca^2+^ exchanger, which is distinct from the plasmalemmal Na^+^–Ca^2+^ exchanger (NCX), is that Li^+^ can substitute for Na^+^ [9]. This unique characteristic contributed to identification of NCLX, a gene responsible for the mitochondrial Na^+^–Ca^2+^ exchanger, as will be described in the next section. The stoichiometry (ion-exchange ratio) and the electrogenicity of the mitochondrial Na^+^–Ca^2+^ exchanger were controversial, but it was believed to be electroneutral [62, 63]. Our group clearly demonstrated, by use of permeabilized rat ventricular myocytes, that the mitochondrial Na^+^–Ca^2+^ exchanger is voltage-dependent and electrogenic, which suggests the stoichiometry is >3Na^+^ for one Ca^2+^ [64]. We also predicted by computer simulation that the voltage dependence of the mitochondrial Na^+^–Ca^2+^ exchanger changes, the affinity becoming lower with mitochondrial membrane depolarization [64]. Because of these features, the mitochondrial Na^+^–Ca^2+^ exchanger dynamically changes the exchange mode (forward or reverse) and modulates the mitochondrial Ca^2+^ concentration in a manner dependent on cytosolic Na^+^ concentration and mitochondrial membrane potential. Recently, several molecules which may modulate the mitochondrial Na^+^–Ca^2+^ exchanger have been identified. Gandhi et al. [18] reported that a deficiency of a PINK1, a 581-amino-acid protein consisting of a mitochondrial targeting motif and a serine/threonine kinase domain homologous with that of the Ca^2+^/calmodulin family, causes impairment of mitochondrial Ca^2+^ efflux via the mitochondrial Na^+^–Ca^2+^ exchanger. Mutations in the PINK1 gene are known to cause autosomal recessive Parkinson’s disease [18]. Da Cruz et al. [65] showed that a stomatin-like protein 2 (SLP-2), a novel member of the stomatin superfamily found in several types of human tumour, negatively modulates mitochondrial Na^+^–Ca^2+^ exchange activity in HeLa cells, regulating the capacity of mitochondria to store Ca^2+^. Although detailed mechanisms underlying the regulation of mitochondrial Na^+^–Ca^2+^ exchange activity by these proteins have not yet been clarified, recent identification of NCLX as a mitochondrial Na^+^–Ca^2+^ exchanger will surely accelerate understanding of the mechanisms.

#### Cloning, tissue distribution, and cellular localization of NCLX

In 2004, two independent research groups reported the cloning of a new transporter mediating Na^+^–Ca^2+^ exchange, which was subsequently identified as a mitochondrial Na^+^–Ca^2+^ exchanger [66, 67]. Cai and Lytton employed a bioinformatics-based search of the GenBank™ database using a conserved amino acid sequence of the α-repeat regions of the K^+^-dependent Na^+^–Ca^2+^ exchanger (NCKX) gene family 2 (NCKX2) [66]. The amino acid sequence of the identified clone was divergent from that of NCX and NCKX family members, but was slightly closer to that of NCKX. The clone was therefore named “NCKX6”. They also found an alternative spliced isoform of mouse NCKX6. Although the long isoform was retained in the ER fraction and was not functional when heterologously expressed in HEK293 cells, the short isoform was targeted in the plasma membrane and had K^+^-dependent Na^+^–Ca^2+^ exchange activity. Very soon after the publication by Cai and Lytton [66], Sekler’s group found the same clone during a search for the Na^+^–Zn^2+^ exchanger gene [67]. In contrast with the report by Cai and Lytton [66], both long and short clones had K^+^-independent Na^+^–Ca^2+^ exchange activity and did not have Na^+^–Zn^2+^ exchange activity when heterologously expressed in HEK293 cells. They also found that Li^+^ can substitute for Na^+^ to release Ca^2+^ from the cells, so they named the clone “NCLX”. The discrepant results obtained by the two groups might be a result of different experimental conditions, i.e. Cai and Lytton [66] used Li^+^ as substituent for Na^+^ to examine K^+^-dependent Na^+^–Ca^2+^ exchange activity. This might have affected the responsiveness of the clone. In addition, FLAG epitope might have interfered with the sorting system in the Cai and Lytton [66] experiments. Anyway, both groups found that NCKX6/NCLX had Na^+^–Ca^2+^ exchange activity and broad tissue distribution, for example heart, pancreas, skeletal muscle, stomach, spleen, and brain.

It took another 6 years, however, for Sekler’s group to discover that NCKX/NCLX is the long-sought mitochondrial Na^+^–Ca^2+^exchanger. In 2010, Sekler’s group beautifully demonstrated that NCLX was located in mitochondria and its characteristics corresponded well to those of the mitochondrial Na^+^–Ca^2+^ exchanger [10]. Direct measurements of mitochondrial Ca^2+^ by use of the mitochondria-targeted Ca^2+^ sensing protein Pericam-mito [68] clearly showed that Ca^2+^ efflux from mitochondria was accelerated by overexpressing NCLX in an Na^+^ and Li^+^-dependent manner and was decelerated by knocking down NCLX. The fact that Li^+^ can substitute for Na^+^ in releasing Ca^2+^ from mitochondria corresponds well to the unique characteristic of mitochondrial Na^+^–Ca^2+^ exchange. This NCLX-mediated Ca^2+^ efflux from mitochondria was inhibited by the mitochondrial Na^+^–Ca^2+^ exchange inhibitor CGP-37157. It was also shown that mitochondrial Na^+^, measured by use of the mitochondrial Na^+^-sensing dye CoroNa Red, increased during the mitochondrial Ca^2+^ efflux phase, and the rate became much faster with NCLX overexpression. These results confirm that NCLX is the gene responsible for the mitochondrial Na^+^–Ca^2+^ exchanger. Since then, information about the involvement of NCLX in the physiological and pathophysiological functions of different types of cell has accumulated. We will describe, in detail, recent findings on the roles of NCLX in pancreatic β-cells, astrocytes, B lymphocytes, and cardiomyocytes. Experimental results from NCLX knockout/knockdown in each type of cell are summarized in Table 1.

**Table 1:** Effects of NCLX reduction on cellular functions

	Mitochondrial Ca^2+^ at rest	Mitochondrial Ca^2+^ transient	Cytosolic Ca^2+^ transient	SOCE	ATP production	Others
Pancreatic β-cells ([70, 71])	Increased	Faster rise slower decline	Smaller	N.D.	Unchanged at steady state. Slightly accelerated during stimulation	Reduced insulin secretion
Astrocytes ([77])	Increased	Faster rise slower decline	Smaller	Smaller and slower	N.D.	Reduced glutamate release Impaired wound healing Impaired proliferation
B lymphocytes ([44, 45, 84])	N.D.	N.D.	Diminished cytosolic Ca^2+^ increase after BCR stimulation	Smaller and slower	N.D.	Increased apoptosis Reduced chemotaxis
Cardiomyocytes ([46, 102])	Increased	N.D.	Slower upstroke	N.D.	Unaffected	Cycle length prolongation

*N.D.* not determined

#### NCLX in pancreatic β-cells (Fig. 4a)

When the plasma glucose level increases, ATP production in pancreatic β-cells increases. ATP-dependent K^+^ channels consequently close, resulting in depolarization of the plasma membrane. Subsequently, voltage-gated Ca^2+^ channels open, causing Ca^2+^ influx into the cell, and exocytosis of insulin granules occurs [69]. In addition to the metabolic roles of mitochondria in the regulation of insulin secretion [70, 71], Tarasov et al. and Nita et al. [72, 73] demonstrated the contribution of NCLX to the function of pancreatic β-cells by use of the pancreatic β cell line MIN6 and mouse pancreatic primary β-cells. By use of pericam-mito to directly sense the mitochondrial Ca^2+^, Nita et al. [73] found that membrane depolarization-mediated Ca^2+^ influx, ATP-mediated ER Ca^2+^ depletion, and treatment with 20 mM glucose all caused a mitochondrial Ca^2+^ transient. In all cases, silencing of NCLX by use of siRNA or dominant negative mutant resulted in a more rapid increase in mitochondrial Ca^2+^ and slower mitochondrial Ca^2+^ decline. The resting mitochondrial Ca^2+^ level was higher in NCLX knockdown cells. These results not only suggest that NCLX functions as a mitochondrial Na^+^–Ca^2+^ exchanger, but also show that Ca^2+^ efflux via NCLX is already activated at the early phase of mitochondrial Ca^2+^ influx. Interestingly, silencing NCLX caused a smaller and slower cytosolic Ca^2+^ increase induced by membrane depolarization or by treatment with 20 mM glucose. Furthermore, NCLX knockdown was followed by a delay in glucose-dependent insulin secretion, whereas ATP production was little affected. These results indicate that NCLX is a critical component of the Ca^2+^ crosstalk between mitochondria and the plasma membrane in modulation of the insulin secretion pattern. Tarasov et al. obtained results essentially similar to those of Nita et al. [73] in repetitively depolarized MIN6 cells with regard to NCLX function. In addition, Tarasov et al. [72] found that glucose-induced mitochondrial Ca^2+^ response and subsequent ATP/ADP increase was impaired under the glucolipotoxic conditions often observed with type 2 diabetes. Because mRNA expression levels of MCU and NCLX were unaltered, it was suggested that changes in the intracellular distribution of mitochondria [74] were involved in this altered Ca^2+^ dysfunction and ATP production. In 2013, Proverbio et al. [75] used a multi-step screening strategy to identify genes related to congenital hyperinsulinism, characterized by severe hypoglycaemia as a result of inappropriate insulin secretion by pancreatic β-cells. They found a mutation in the NCLX-encoding SLC24A6 gene, which results in amino acid change at position 564 from tyrosine to histidine. Because this position resides in the middle of putative transmembrane domain 12 and is conserved among a variety of species, including chimpanzee, mouse, rat, cattle, chicken, and zebrafish, it is possible that the mutation causes a functional change of NCLX. Further analysis is required to clarify how NCLX is involved in the pathophysiological condition of pancreatic β-cells.

**Fig. 4:**
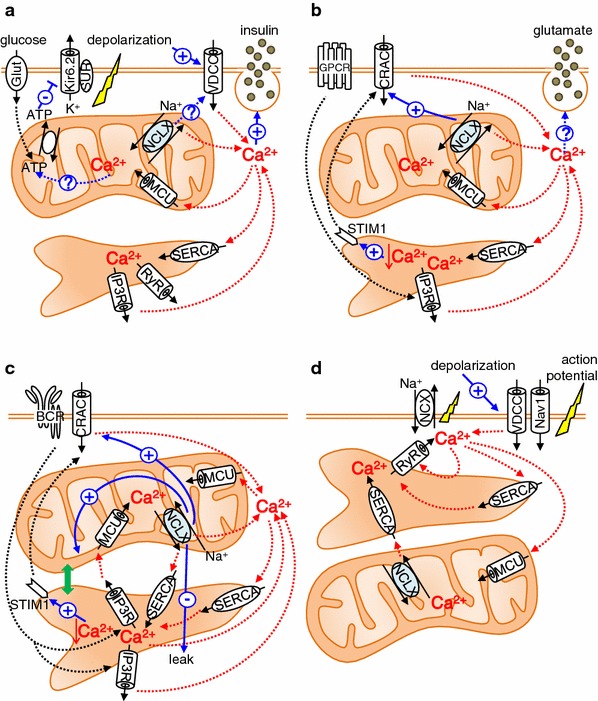
Roles of NCLX in pancreatic β-cells (**a**), astrocytes (**b**), B lymphocytes (**c**), and cardiomyocytes (**d**). See text for details

#### NCLX in astrocytes (Fig. 4b)

Brain is another tissue highly expressing NCLX. Among the different kinds of cell constituting the brain, astrocytes constitute approximately half the volume. They express a large number of G protein-coupled receptors (GPCRs), linked to a diverse array of intracellular cascades including elevation of intracellular Ca^2+^. It is also well known that astrocytes release neurotransmitters called gliotransmitters, for example glutamate, ATP and d-serine, which bind to neuronal receptors to modulate synaptic transmission and activity [76, 77]. Thus astrocytes not only interact with neuronal activity but also modulate this activity via gliotransmitters. Although still controversial, several reports suggest that release of gliotransmitters by astrocytes depends on elevation of cytosolic Ca^2+^ [77, 78]. The increase of cytosolic Ca^2+^ in astrocytes occurs via the phospholipase C (PLC)/IP_3_ pathway. That is, upon GPCR activation, PLC hydrolyses the membrane lipid phosphatidylinositol 4,5-bisphosphate to generate diacylglycerol and IP_3_, activating the IP_3_ receptor (IP_3_R) and releasing Ca^2+^ from the ER. Parnis et al. [79], by use of a mouse astrocyte culture, examined how NCLX is involved in Ca^2+^ dynamics and the release of gliotransmitters. They used pericam-mito to sense mitochondrial Ca^2+^ and found that the resting mitochondrial Ca^2+^ level increased, and that the rise and decline of mitochondrial Ca^2+^ transients caused by extracellular ATP application became faster and slower, respectively, on knocking down NCLX. These results were very similar to those observed for pancreatic β-cells [73]. Interestingly, NCLX knockdown reduced Ca^2+^ entry into the cytosol both from extracellular space and from the ER, but the effect on the former was stronger. Detailed analysis revealed that NCLX knockdown impairs store-operated Ca^2+^ entry (SOCE), indicative of strong Ca^2+^ crosstalk between mitochondria and the plasma membrane, as in pancreatic β-cells. NCLX knockdown also significantly reduced such processes as exocytotic glutamate release, in vitro wound closure, and proliferation, which may be regulated in a Ca^2+^-dependent manner [79].

#### NCLX in B lymphocytes (Fig. 4c)

**Fig. 5:**
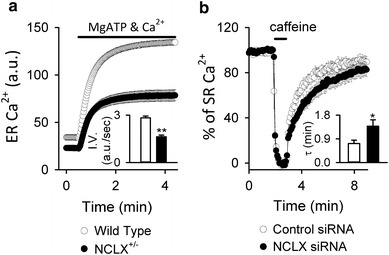
Modulation of SERCA activity by NCLX reduction in (**a**) DT40 B lymphocytes and (**b**) HL-1 cardiomyocytes. **a** ER Ca^2+^ uptake by wild type and NCLX^+/−^ DT40 cells. ER Ca^2+^ uptake were activated by applying 0.1 mM MgATP and 100 nM Ca^2+^ in Mag Fluo-4-loaded and permeabilized cells under conditions in which mitochondrial respiration was intact. The *bar graph* depicts the initial velocity of the ER Ca^2+^ increase. Data are mean ± SEM of independent recordings. Modified from Kim et al. [44]. **b** SR Ca^2+^ reuptake by HL-1 cardiomyocytes. Plasmid harbouring Cameleon D1ER, an indicator of SR Ca^2+^, was co-transfected with control (*white*) or NCLX siRNA (*black*) into HL-1 cardiomyoyctes. After emptying Ca^2+^ in SR with 10 mM caffeine, recovery of SR Ca^2+^ was measured. The *bar graph* depicts the recovery time constant *τ*, showing that SR Ca^2+^ reuptake was slower in NCLX knockdown cells. **p < 0.01, *p < 0.05. Modified from Takeuchi et al. [46]

Activation of B lymphocytes by antigens is followed by an increase in cytosolic Ca^2+^, resulting in rapid proliferation and differentiation or apoptosis, depending on the differentiation stage of the cells [80]. Upon binding of the antigen to the B cell surface receptor (BCR), IP_3_ increases and facilitates Ca^2+^ release from the IP_3_R on the ER membrane. Ca^2+^ release from the ER causes the initial cytosolic Ca^2+^ increase after receptor activation. Subsequent depletion of Ca^2+^ in the ER causes translocation of stromal interaction molecule 1 (STIM1) to the vicinity of plasmalemma, inducing a sustained and oscillatory cytosolic Ca^2+^ increase by activation of SOCE through CRAC channels encoded by ORAI1 [81, 82]. We discovered that NCLX is crucial in this antigen receptor -mediated Ca^2+^ signalling from a combined study of mathematical simulations and NCLX knockout/knockdown in DT40 and A20 B lymphocytes [46, 47]. NCLX reduction greatly reduced cytosolic Na^+^-dependent mitochondrial Ca^2+^ efflux in saponin-permeabilized cells loaded with the mitochondrial Ca^2+^-sensitive dye Rhod-2, confirming that NCLX is responsible for mitochondrial Na^+^–Ca^2+^ exchange in B lymphocytes. Interestingly, mitochondrial membrane potential, evaluated by JC-1 staining, was more depolarized and DNA fragmentation (sub G1) was increased in NCLX knockout cells, suggesting that the progress of apoptosis was accelerated in NCLX knockout cells. Mathematical model revealed that NCLX inhibition reduces basal ER Ca^2+^ content and suppresses BCR-mediated cytosolic Ca^2+^ rise. These predictions were validated by experiments. i.e., ER Ca^2+^ content decreased and the cytosolic increase of Ca^2+^ was diminished in NCLX knockout/knockdown cells after the BCR activation. Comparable with the results obtained by use of astrocytes, SOCE activity in B lymphocytes was also diminished by silencing NCLX, probably further contributing to impairment of the cytosolic Ca^2+^ increase after BCR activation [47]. The reduction in ER Ca^2+^ content was a result of impaired Ca^2+^ supply from mitochondria via NCLX, because ER Ca^2+^ uptake via the ER Ca^2+^ pump SERCA was decelerated by NCLX reduction when mitochondrial respiration was intact (Fig. 5a) whereas it was comparable when mitochondrial respiration was disturbed [47]. Interesting findings were that NCLX reduction resulted in impaired co-localization of mitochondria with ER and augmented ER Ca^2+^ leak. Although the cause remains unresolved, NCLX may be associated with the tethering proteins connecting ER and mitochondria [83, 84], and reduction of NCLX may weaken mitochondria–ER interactions. Alternatively, mitochondrial depolarization induced by NCLX knockout/knockdown might result in elimination of the impaired mitochondria by mitophagy [85]. Taken together, our results not only show that NCLX is involved in the crosstalk between mitochondria and the plasma membrane, as revealed by SOCE regulation, but also indicate that NCLX-mediated crosstalk between mitochondria and ER Ca^2+^ dynamics is crucial for physiological functioning of B lymphocytes; i.e. a response to BCR activation. Very recently we found that silencing NCLX reduced the chemotaxis of B lymphocytes triggered by chemokine CXCL12, although the mechanism is still unclear [86]. Although further analysis is needed, it is likely that NCLX is important in regulation of immune systems.

#### NCLX in cardiomyocytes (Fig. 4d)

The heart is one of the most studied organs for investigation of the characteristics of the mitochondrial Na^+^–Ca^2+^ exchanger, which serves as a major Ca^2+^ release system [87, 88]. The roles of the mitochondrial Na^+^–Ca^2+^ exchanger in cardiac energetics have been widely investigated. For example, activation of the mitochondrial Na^+^–Ca^2+^ exchanger by increasing cytosolic Na^+^ causes a decrease of mitochondrial Ca^2+^, resulting in an imbalance in energy demand and supply or in an increase of ROS production [89, 90]. In contrast, the contribution of the mitochondrial Na^+^–Ca^2+^ exchanger to cytosolic Ca^2+^ transients and to action potential generation has been regarded as negligible, because the contribution of mitochondrial Ca^2+^ uptake to Ca^2+^ release from cardiomyocytes is as low as 1–2 % [91]. However, we recently discovered that NCLX participates in modulation of action potential configuration and in regulation of the automaticity of HL-1, a spontaneously beating cardiac cell line originating from mouse atrial myocytes [48]. The expression patterns of ion channels and transporters in HL-1 cells are similar to those in adult atrial myocytes, except that HL-1 cells highly express the T-type Ca^2+^ channel (*I*_CaT_) and the hyperpolarization-activated cation channel (*I*_ha_), which are known to be involved in the automaticity of cardiac pacemaker sinoatrial (SA) node cells [92–95]. NCLX reduction using siRNA reduced NCLX protein expression by ~50 %, resulting in a ~50 % reduction of the rate of cytosolic Na^+^-dependent mitochondrial Ca^2+^ efflux, confirming that NCLX is responsible for mitochondrial Na^+^–Ca^2+^ exchange in HL-1 cells. Mitochondrial Ca^2+^ content, evaluated as the Ca^2+^ chelation-responsive fraction of the intensity of mitochondrial Ca^2+^ sensor pCase12-mito, was larger in NCLX knockdown cells. Although beat-to-beat change of mitochondrial Ca^2+^ was not observed in HL-1 cells, the result indicated that NCLX contributes to the steady state mitochondrial Ca^2+^ content in intact HL-1 cells. Cellular energetics seemed to be unaffected by NCLX knockdown, because there were no differences in cellular ATP content, mitochondrial membrane potential, or mitochondrial ROS between control cells and NCLX knockdown cells. This may be because protein expression of NCLX knockdown using siRNA was reduced by 50 % only.

An interesting finding was that NCLX knockdown caused marked prolongation of the cycle length of spontaneous action potentials and Ca^2+^ transients [48]. Kinetic analysis of electrophysiological data and Ca^2+^ transients obtained by line scanning cells loaded with cytosolic Ca^2+^ indicator Fluo-4 revealed that NCLX knockdown slowed the upstrokes of both action potentials and cytosolic Ca^2+^ transients. SR Ca^2+^ dynamics, which is known to contribute to the upstroke of cytosolic Ca^2+^ transients, was evaluated by use of ER/SR Ca^2+^ FRET protein cameleon D1ER [96]. As a result, SR Ca^2+^ content of NCLX knockdown cells was smaller and SR Ca^2+^ reuptake rate was slower (Fig. 5b), suggesting that the NCLX reduction-mediated prolongation of cycle length is related to compromised SR Ca^2+^ dynamics. The mechanisms underlying the NCLX reduction-mediated prolongation of cycle length was further studied with a newly constructed mathematical model of HL-1 cells [48]. The HL-1 cell model well reproduces the spontaneous generation of action potentials and Ca^2+^ transients, and the prolongation of the cycle length induced by knocking down NCLX. The model analysis indicated that automaticity of HL-1 cells is determined by spontaneous Ca^2+^ leak from the SR. Simulation of NCLX reduction showed that Ca^2+^ supply from the mitochondria to the SR decreased to slow down the rate of spontaneous Ca^2+^ leak from the SR. The timing of Ca^2+^-induced Ca^2+^ release (CICR), activation of the inward current of the plasma membrane NCX (*I*_NCX_), and thus the timing of activation of voltage-dependent Na^+^ current (*I*_Na_) and voltage-dependent T and L type Ca^2+^ channels (*I*_CaT_ and *I*_CaL_) was thus delayed, resulting in prolongation of the cycle length. Taken together, our combined experiments and simulations indicated that NCLX regulates the rhythmicity of HL-1 cells via crosstalk between mitochondria and SR Ca^2+^ dynamics. Considering that NCLX reduction resulted in modification of plasma membrane NCX activity, NCLX may also be indirectly involved in mitochondria–plasma membrane Ca^2+^ crosstalk in HL-1 cells. Interestingly, Opuni and Reeves reported the functional coupling of mitochondrial function, possibly mitochondrial Na^+^–Ca^2+^ exchange activity, and plasma membrane NCX activity in Chinese hamster ovary cells stably transfected with bovine cardiac NCX [97]. Further analysis is necessary to elucidate the interaction of mitochondrial NCLX and plasma membrane NCX. Because HL-1 cells are derived from atrial myocytes, which have no automaticity under physiological conditions, NCLX may be involved in the abnormal automaticity of atria, for example atrial flutter or atrial ectopic tachycardia. It may also be possible that occurrence of arrhythmia in patients with mitochondrial disease [98, 99] is caused by abnormal NCLX function. Further analysis is required to clarify the involvement of NCLX in these arrhythmias.

Whether NCLX participates in the automaticity of normal pacemaker cells, sinoatrial (SA) node cells, is a big issue. Recently, Yaniv et al. [100] reported that mitochondrial Na^+^–Ca^2+^ exchange inhibitor CGP-37157 slowed the generation of automaticity of rabbit SA node cells, suggesting that mitochondrial Na^+^–Ca^2+^ exchange is involved in generation of the automaticity of SA node cells. However, because CGP-37157 also blocks *I*_CaL_, which is related to generation of the automaticity of SA node cells, a nonspecific effect of CGP-37157 on *I*_CaL_ cannot be ignored. In addition, the automaticity of the SA node cells they used significantly depends on spontaneous and local subsarcolemmal Ca^2+^ releases from SR, the so called “Ca^2+^ clock” mechanism [101]. However, this automaticity mechanism has been controversial. It has been accepted for a long time that *I*_ha_ and a variety of other inward membrane currents determine the automaticity [102–104]. This mechanism is called the “membrane clock”. Whether NCLX contributes to the automaticity of all types of SA node cells, including the cells driven by “membrane clock”, must be studied.

We examined the contribution of NCLX using mathematical models of SA node cells [105]. In the original SA node model developed by Yaniv et al. [100], complete reduction of mitochondrial Na^+^–Ca^2+^ exchange resulted in prolongation of cycle length by 2.7 % only. The larger effect reported by Yaniv et al. [100] is probably because of their simultaneous reduction of the amplitude factor of SERCA. To further test the contribution of NCLX to generation of the automaticity of SA node cell models, we incorporated our model of mitochondrial Ca^2+^ dynamics into two representative SA node cell models: a membrane clock model by Himeno et al. [102, 103] and a Ca^2+^ clock model by Maltsev and Lakatta [101]. In both models, NCLX reduction reduced the SR Ca^2+^ content, supporting the idea of Ca^2+^ communication between mitochondria and the SR in SA node cells. However, the effect on the automaticity was different between the models. The cycle length was prolonged in the Maltsev and Lakatta model whereas it was shortened in the Himeno model [105]. Furthermore, model analysis revealed that cytosolic Na^+^ and Na^+^-permeable inward current (sustained inward current *I*_st_) [106] are crucial factors distinguishing the effect of NCLX on pace-making in the two models. In the Himeno model, NCLX reduction reduced cytosolic Ca^2+^, which decreased the inward *I*_NCX_ thus reducing the cytosolic Na^+^ concentration. This increased the amplitude of the inward *I*_st_ and overcame the decrease of inward *I*_NCX_, accelerating the firing rate. In contrast, the amplitude of *I*_st_ is set smaller in the Maltsev and Lakatta model so that NCLX reduction only reduces inward *I*_NCX_ and slows diastolic depolarization, decelerating the firing rate [105]. Accordingly, it is likely that the Ca^2+^ communication between mitochondria and the SR via NCLX functions also in SA node cells. However, it is necessary to investigate quantitatively how much the ‘‘Ca^2+^ clock’’ mechanism, cytoplasmic Na^+^, and *I*_st_ contribute to SA node automaticity.

### Mitochondrial H^+^–Ca^2+^ exchanger

H^+^–Ca^2+^ exchange has a dominant effect on release of Ca^2+^ from mitochondria in tissues in which mitochondrial Na^+^–Ca^2+^ exchange activity is low, for example the liver, kidney, lung, and smooth muscle [61]. The H^+^–Ca^2+^ exchanger also occurs in the heart, though the activity is weak [61, 107].

The stoichiometry of the H^+^–Ca^2+^ exchanger is regarded as 2H^+^ for 1Ca^2+^, being electroneutral. However, because the rate of efflux via the H^+^–Ca^2+^ exchanger decreases with increasing pH gradient in rat isolated liver mitochondria, it is suggested that the mechanism is not a passive Ca^2+^ for 2H^+^ exchanger, but an active Ca^2+^ for 2H^+^ exchanger [108].

The molecular identity of the mitochondrial H^+^–Ca^2+^ exchanger is still controversial. The candidate is the Letm1 (leucine–zipper–EF hand-containing transmembrane region). Jiang et al. [11] conducted high-throughput RNA interference screening of *Drosophila* genes and identified a gene affecting mitochondrial Ca^2+^ and H^+^, the human homolog of which is Letm1. By using digitonin-permeabilized S2 or 293 cells expressing mitochondrial Ca^2+^ sensor protein pericam, or by using purified Letm1 reconstituted in liposomes, they found that Letm1 mediates H^+^–Ca^2+^ exchange. However, because a drastic reduction of mitochondrial Ca^2+^ uptake was observed when Letm1 protein expression was suppressed, and because H^+^–Ca^2+^ exchange via Letm1 was sensitive to an inhibitor of mitochondrial Ca^2+^ influx, ruthenium red, Letm1 is regarded as mediating Ca^2+^ influx into mitochondria, at least at low cytosolic Ca^2+^ level. This idea was confirmed by subsequent work by Jiang et al. [109] in which Letm1 knockdown in 293 cells resulted in dramatically reduced mitochondrial Ca^2+^ content. In the same work they produced Letm1 knockout mice and found that Letm1 homozygous knockout mice were embryonic lethal and so were half of the heterozygous knockout mice. The surviving mice had altered glucose metabolism, impaired control of brain ATP levels, and increased seizure activity, suggesting involvement of Letm1 in the pathology of Wolf–Hirschhorn syndrome, in which Letm1 is known to be one of the genes deleted [110]. Nowikovsky et al. [111] analysed theoretically the direction of Ca^2+^ flux through the mitochondrial membrane in energized mitochondria with different H^+^:Ca^2+^ stoichiometry. They proposed that Ca^2+^ enters mitochondria with 1H^+^:1Ca^2+^ whereas Ca^2+^ leaves mitochondria with 2H^+^:1Ca^2+^ or 3H^+^:1Ca^2+^ stoichiometry under physiological respiration conditions. Tsai et al. [112] reported that Letm1 mediates the electroneutral 2H^+^:1Ca^2+^ antiport and is insensitive to ruthenium red, by using Letm1 reconstituted proteoliposome. These results combined with the theoretical analysis by Nowikovsky et al. [111] strongly suggest that Letm1 is the long-awaited molecular identity of the mitochondrial H^+^–Ca^2+^ exchanger. There is, currently, no reasonable explanation of the discrepancy of the sensitivity of Letm1 to ruthenium red. We independently found that a substantial part of the Ca^2+^ efflux from mitochondria was independent of cytosolic Na^+^ when mitochondria were loaded with a lower concentration of Ca^2+^ than when examining Na^+^–Ca^2+^ exchange activity using A20 B lymphocytes [46]. This fraction, which probably represents the H^+^–Ca^2+^ exchange system, was sensitive to ruthenium red. Further analysis is necessary to determine whether Letm1 is, indeed, the H^+^–Ca^2+^ exchanger mediating Ca^2+^ extrusion from mitochondria.

## Summary

Ca^2+^, not only inside mitochondria but also released from mitochondria, is crucially important in regulating a variety of cellular physiological functions. Identification of the molecules responsible for the pathways of Ca^2+^ release enables us to isolate the contributions of these molecules. For example, NCLX has been shown to participate in insulin secretion in pancreatic β-cells, glutamate release in astrocytes, Ca^2+^ responsiveness to BCR stimulation in B lymphocytes, and generation of the spontaneous rhythmicity of cadiomyocytes, via Ca^2+^ crosstalk between mitochondria and the plasma membrane and/or between mitochondria and the ER/SR (Fig. 4). However, many issues remain unanswered. One is the question of whether mitochondrial Ca^2+^ release proteins are indeed located in the tethering spots between mitochondria and the ER/SR or between mitochondria and the plasma membrane. Another is the contribution of the mitochondrial Ca^2+^ release system to mitochondrial energetics. Distinct phenotypes of NCLX knockdown cells related to mitochondrial energetics in cardiomyocytes and in pancreatic β-cells have not been observed, while Letm1 knockout mice had impaired mitochondrial energetics. Complete knockout of NCLX in mice or cells will clarify the matter.

## References

[1] Bernardi P (1999). Mitochondrial transport of cations: channels, exchangers, and permeability transition. Physiol Rev.

[2] Murgia M, Giorgi C, Pinton P, Rizzuto R (2009). Controlling metabolism and cell death: at the heart of mitochondrial calcium signalling. J Mol Cell Cardiol.

[3] Celsi F, Pizzo P, Brini M, Leo S, Fotino C, Pinton P, Rizzuto R (2009). Mitochondria, calcium and cell death: a deadly triad in neurodegeneration. Biochim Biophys Acta.

[4] De Stefani D, Raffaello A, Teardo E, Szabò I, Rizzuto R (2011). A forty-kilodalton protein of the inner membrane is the mitochondrial calcium uniporter. Nature.

[5] Baughman JM, Perocchi F, Girgis HS, Plovanich M, Belcher-Timme CA, Sancak Y, Bao XR, Strittmatter L, Goldberger O, Bogorad RL, Koteliansky V, Mootha VK (2011). Integrative genomics identifies MCU as an essential component of the mitochondrial calcium uniporter. Nature.

[6] Niescier RF, Chang KT, Min KT (2013). Miro, MCU, and calcium: bridging our understanding of mitochondrial movement in axons. Front Cell Neurosci.

[7] Patron M, Raffaello A, Granatiero V, Tosatto A, Merli G, De Stefani D, Wright L, Pallafacchina G, Terrin A, Mammucari C, Rizzuto R (2013). The mitochondrial calcium uniporter (MCU): molecular identity and physiological roles. J Biol Chem.

[8] Marchi S, Pinton P (2014). The mitochondrial calcium uniporter complex: molecular components, structure and physiopathological implications. J Physiol.

[9] Carafoli E, Tiozzo R, Lugli G, Crovetti F, Kratzing C (1974). The release of calcium from heart mitochondria by sodium. J Mol Cell Cardiol.

[10] Palty R, Silverman WF, Hershfinkel M, Caporale T, Sensi SL, Parnis J, Nolte C, Fishman D, Shoshan-Barmatz V, Herrmann S, Khananshvili D, Sekler I (2010). NCLX is an essential component of mitochondrial Na^+^/Ca^2+^ exchange. Proc Natl Acad Sci U S A.

[11] Jiang D, Zhao L, Clapham DE (2009). Genome-wide RNAi screen identifies Letm1 as a mitochondrial Ca^2+^/H^+^ antiporter. Science.

[12] Rowland AA, Voeltz GK (2012). Endoplasmic reticulum-mitochondria contacts: function of the junction. Nat Rev Mol Cell Biol.

[13] van Vliet AR, Verfaillie T, Agostinis P (2014) New functions of mitochondria associated membranes in cellular signaling. Biochim Biophys Acta (in press). doi:10.1016/j.bbamcr.2014.03.009 10.1016/j.bbamcr.2014.03.009 24642268

[14] McCormack JG, Halestrap AP, Denton RM (1990). Role of calcium ions in regulation of mammalian intramitochondrial metabolism. Physiol Rev.

[15] Jo H, Noma A, Matsuoka S (2006). Calcium-mediated coupling between mitochondrial substrate dehydrogenation and cardiac workload in single guinea-pig ventricular myocytes. J Mol Cell Cardiol.

[16] Satrústegui J, Pardo B, Del Arco A (2007). Mitochondrial transporters as novel targets for intracellular calcium signalin. Physiol Rev.

[17] Chacon E, Acosta D (1991). Mitochondrial regulation of superoxide by Ca^2+^: an alternate mechanism for the cardiotoxicity of doxorubicin. Toxicol Appl Pharmacol.

[18] Gandhi S, Wood-Kaczmar A, Yao Z, Plun-Favreau H, Deas E, Klupsch K, Downward J, Latchman DS, Tabrizi SJ, Wood NW, Duchen MR, Abramov AY (2009). PINK1-associated Parkinson’s disease is caused by neuronal vulnerability to calcium-induced cell death. Mol Cell.

[19] Harris DA (1993). Regulation of the mitochondrial ATP synthase in rat heart. Biochem Soc Trans.

[20] Scholz TD, Balaban RS (1994). Mitochondrial F1-ATPase activity of canine myocardium: effects of hypoxia and stimulation. Am J Physiol.

[21] Crompton M (1999). The mitochondrial permeability transition pore and its role in cell death. Biochem J.

[22] Bernardi P, Krauskopf A, Basso E, Petronilli V, Blachly-Dyson E, Di Lisa F, Forte MA (2006). The mitochondrial permeability transition from in vitro artifact to disease target. FEBS J.

[23] Rizzuto R, De Stefani D, Raffaello A, Mammucari C (2012). Mitochondria as sensors and regulators of calcium signalling. Nat Rev Mol Cell Biol.

[24] Wang X (2001). The expanding role of mitochondria in apoptosis. Genes Dev.

[25] Hajnóczky G, Davies E, Madesh M (2003). Calcium signaling and apoptosis. Biochem Biophys Res Commun.

[26] Lemasters JJ, Theruvath TP, Zhong Z, Nieminen AL (2009). Mitochondrial calcium and the permeability transition in cell death. Biochim Biophys Acta.

[27] Dedkova EN, Blatter LA (2009). Characteristics and function of cardiac mitochondrial nitric oxide synthase. J Physiol.

[28] Tidow H, Nissen P (2013). Structural diversity of calmodulin binding to its target sites. FEBS J.

[29] Gilabert JA (2012). Cytoplasmic calcium buffering. Adv Exp Med Biol.

[30] Lawrie AM, Rizzuto R, Pozzan T, Simpson AW (1996). A role for calcium influx in the regulation of mitochondrial calcium in endothelial cells. J Biol Chem.

[31] Babcock DF, Herrington J, Goodwin PC, Park YB, Hille B (1997). Mitochondrial participation in the intracellular Ca^2+^ network. J Cell Biol.

[32] Park MK, Ashby MC, Erdemli G, Petersen OH, Tepikin AV (2001). Perinuclear, perigranular and sub-plasmalemmal mitochondria have distinct functions in the regulation of cellular calcium transport. EMBO J.

[33] Giacomello M, Drago I, Bortolozzi M, Scorzeto M, Gianelle A, Pizzo P, Pozzan T (2010). Ca^2+^ hot spots on the mitochondrial surface are generated by Ca^2+^ mobilization from stores, but not by activation of store-operated Ca^2+^ channels. Mol Cell.

[34] Hoth M, Fanger CM, Lewis RS (1997). Mitochondrial regulation of store-operated calcium signaling in T lymphocytes. J Cell Biol.

[35] Glitsch MD, Bakowski D, Parekh AB (2002). Store-operated Ca^2+^ entry depends on mitochondrial Ca^2+^ uptake. EMBO J.

[36] Csordás G, Renken C, Varnai P, Walter L, Weaver D, Buttle KF, Balla T, Mannella CA, Hajnóczky G (2006). Structural and functional features and significance of the physical linkage between ER and mitochondria. J Cell Biol.

[37] Friedman JR, Lackner LL, West M, DiBenedetto JR, Nunnari J, Voeltz GK (2011). ER tubules mark sites of mitochondrial division. Science.

[38] Sharma VK, Ramesh V, Franzini-Armstrong C, Sheu SS (2000). Transport of Ca^2+^ from sarcoplasmic reticulum to mitochondria in rat ventricular myocytes. J Bioenerg Biomembr.

[39] Csordás G, Várnai P, Golenár T, Roy S, Purkins G, Schneider TG, Balla T, Hajnóczky G (2010). Imaging interorganelle contacts and local calcium dynamics at the ER-mitochondrial interface. Mol Cell.

[40] Rizzuto R (1998). Close contacts with the endoplasmic reticulum as determinants of mitochondrial Ca^2+^ responses. Science.

[41] Mendes CC, Gomes DA, Thompson M, Souto NC, Goes TS, Goes AM, Rodrigues MA, Gomez MV, Nathanson MH, Leite MF (2005). The type III inositol 1,4,5-trisphosphate receptor preferentially transmits apoptotic Ca^2+^ signals into mitochondria. J Biol Chem.

[42] Szabadkai G, Bianchi K, Várnai P, De Stefani D, Wieckowski MR, Cavagna D, Nagy AI, Balla T, Rizzuto R (2006). Chaperone-mediated coupling of endoplasmic reticulum and mitochondrial Ca^2+^ channels. J Cell Biol.

[43] Arnaudeau S, Kelley WL, Walsh JV, Demaurex N (2001). Mitochondria recycle Ca^2+^ to the endoplasmic reticulum and prevent the depletion of neighboring endoplasmic reticulum regions. J Biol Chem.

[44] Malli R, Frieden M, Trenker M, Graier WF (2005). The role of mitochondria for Ca^2+^ refilling of the endoplasmic reticulum. J Biol Chem.

[45] Poburko D, Liao CH, van Breemen C, Demaurex N (2009). Mitochondrial regulation of sarcoplasmic reticulum Ca^2+^ content in vascular smooth muscle cells. Circ Res.

[46] Kim B, Takeuchi A, Koga O, Hikida M, Matsuoka S (2012). Pivotal role of mitochondrial Na^+^–Ca^2+^ exchange in antigen receptor mediated Ca^2+^ signalling in DT40 and A20 B lymphocytes. J Physiol.

[47] Kim B, Takeuchi A, Koga O, Hikida M, Matsuoka S (2013). Mitochondria Na^+^–Ca^2+^ exchange in cardiomyocytes and lymphocytes. Adv Exp Med Biol.

[48] Takeuchi A, Kim B, Matsuoka S (2013). The mitochondrial Na^+^–Ca^2+^ exchanger, NCLX, regulates automaticity of HL-1 cardiomyocytes. Sci Rep.

[49] Deluca HF, Engstrom GW (1961). Calcium uptake by rat kidney mitochondria. Proc Natl Acad Sci U S A.

[50] Vasington FD, Murphy JV (1962). Ca^++^ uptake by rat kidney mitochondria and its dependence on respiration and phosphorylation. J Biol Chem.

[51] Fiskum G, Lehninger AL (1979). Regulated release of Ca^2+^ from respiring mitochondria by Ca^2+^/2H^+^ antiport. J Biol Chem.

[52] Gunter TE, Yule DI, Gunter KK, Eliseev RA, Salter JD (2004). Calcium and mitochondria. FEBS Lett.

[53] Carafoli E (2010). The fateful encounter of mitochondria with calcium: how did it happen?. Biochim Biophys Acta.

[54] Malli R, Graier WF (2010). Mitochondrial Ca^2+^ channels: great unknowns with important functions. FEBS Lett.

[55] Kirichok Y, Krapivinsky G, Clapham DE (2004). The mitochondrial calcium uniporter is a highly selective ion channel. Nature.

[56] Perocchi F, Gohil VM, Girgis HS, Bao XR, McCombs JE, Palmer AE, Mootha VK (2010). MICU1 encodes a mitochondrial EF hand protein required for Ca^2+^ uptake. Nature.

[57] Huang G, Vercesi AE, Docampo R (2013). Essential regulation of cell bioenergetics in *Trypanosoma brucei* by the mitochondrial calcium uniporter. Nat Commun.

[58] Pan X, Liu J, Nguyen T, Liu C, Sun J, Teng Y, Fergusson MM, Rovira II, Allen M, Springer DA, Aponte AM, Gucek M, Balaban RS, Murphy E, Finkel T (2013). The physiological role of mitochondrial calcium revealed by mice lacking the mitochondrial calcium uniporter. Nat Cell Biol.

[59] Crompton M, Moser R, Lüdi H, Carafoli E (1978). The interrelations between the transport of sodium and calcium in mitochondria of various mammalian tissues. Eur J Biochem.

[60] Haworth RA, Hunter DR, Berkoff HA (1980). Na^+^ releases Ca^2+^ from liver, kidney and lung mitochondria. FEBS Lett.

[61] Gunter TE, Pfeiffer DR (1990). Mechanisms by which mitochondria transport calcium. Am J Physiol.

[62] Affolter H, Carafoli E (1980). The Ca^2+^–Na^+^ antiporter of heart mitochondria operates electroneutrally. Biochem Biophys Res Commun.

[63] Wingrove DE, Gunter TE (1986). Kinetics of mitochondrial calcium transport. II. A kinetic description of the sodium-dependent calcium efflux mechanism of liver mitochondria and inhibition by ruthenium red and by tetraphenylphosphonium. J Biol Chem.

[64] Kim B, Matsuoka S (2008). Cytoplasmic Na^+^-dependent modulation of mitochondrial Ca^2+^ via electrogenic mitochondrial Na^+^–Ca^2+^ exchange. J Physiol.

[65] Da Cruz S, De Marchi U, Frieden M, Parone PA, Martinou JC, Demaurex N (2010). SLP-2 negatively modulates mitochondrial sodium-calcium exchange. Cell Calcium.

[66] Cai X, Lytton J (2004). Molecular cloning of a sixth member of the K^+^-dependent Na^+^/Ca^2+^ exchanger gene family, NCKX6. J Biol Chem.

[67] Palty R, Ohana E, Hershfinkel M, Volokita M, Elgazar V, Beharier O, Silverman WF, Argaman M, Sekler I (2004). Lithium-calcium exchange is mediated by a distinct potassium-independent sodium-calcium exchanger. J Biol Chem.

[68] Nagai T, Sawano A, Park ES, Miyawaki A (2001). Circularly permuted green fluorescent proteins engineered to sense Ca^2+^. Proc Natl Acad Sci U S A.

[69] Ashcroft FM (2007). ATP-sensitive K^+^ channels and disease: from molecule to malady. Am J Physiol Endocrinol Metab.

[70] Ashcroft SJ, Weerasinghe LC, Randle PJ (1973). Interrelationship of islet metabolism, adenosine triphosphate content and insulin release. Biochem J.

[71] Kennedy HJ (1999). Glucose generates sub-plasma membrane ATP microdomains in single islet β-cells. Potential role for strategically located mitochondria. J Biol Chem.

[72] Tarasov AI, Semplici F, Ravier MA, Bellomo EA, Pullen TJ, Gilon P, Sekler I, Rizzuto R, Rutter GA (2012). The mitochondrial Ca^2+^ uniporter MCU is essential for glucose-induced ATP increases in pancreatic β-cells. PLoS ONE.

[73] Nita II, Hershfinkel M, Fishman D, Ozeri E, Rutter GA, Sensi SL, Khananshvili D, Lewis EC, Sekler I (2012). The mitochondrial Na^+^/Ca^2+^ exchanger upregulates glucose dependent Ca^2+^ signalling linked to insulin secretion. PLoS ONE.

[74] Molina AJ, Wikstrom JD, Stiles L, Las G, Mohamed H, Elorza A, Walzer G, Twig G, Katz S, Corkey BE, Shirihai OS (2009). Mitochondrial networking protects β-cells from nutrient-induced apoptosis. Diabetes.

[75] Proverbio MC, Mangano E, Gessi A, Bordoni R, Spinelli R, Asselta R, Valin PS, Di Candia S, Zamproni I, Diceglie C, Mora S, Caruso-Nicoletti M, Salvatoni A, De Bellis G, Battaglia C (2013). Whole genome SNP genotyping and exome sequencing reveal novel genetic variants and putative causative genes in congenital hyperinsulinism. PLoS ONE.

[76] Agulhon C, Petravicz J, McMullen AB, Sweger EJ, Minton SK, Taves SR, Casper KB, Fiacco TA, McCarthy KD (2008). What is the role of astrocyte calcium in neurophysiology?. Neuron.

[77] Navarrete M, Araque A (2008). Endocannabinoids mediate neuron-astrocyte communication. Neuron.

[78] Serrano A, Haddjeri N, Lacaille JC, Robitaille R (2006). GABAergic network activation of glial cells underlies hippocampal heterosynaptic depression. J Neurosci.

[79] Parnis J, Montana V, Delgado-Martinez I, Matyash V, Parpura V, Kettenmann H, Sekler I, Nolte C (2013). Mitochondrial exchanger NCLX plays a major role in the intracellular Ca^2+^ signaling, gliotransmission, and proliferation of astrocytes. J Neurosci.

[80] Scharenberg AM, Humphries LA, Rawlings DJ (2007). Calcium signalling and cell-fate choice in B cells. Nat Rev Immunol.

[81] Feske S (2007). Calcium signalling in lymphocyte activation and disease. Nat Rev Immunol.

[82] Vig M, Kinet JP (2009). Calcium signaling in immune cells. Nat Immunol.

[83] Pinton P, Giorgi C, Siviero R, Zecchini E, Rizzuto R (2008). Calcium and apoptosis: ER-mitochondria Ca^2+^ transfer in the control of apoptosis. Oncogene.

[84] Csordás G, Hajnóczky G (2009). SR/ER-mitochondrial local communication: calcium and ROS. Biochim Biophys Acta.

[85] Youle RJ, Narendra DP (2011). Mechanisms of mitophagy. Nat Rev Mol Cell Biol.

[86] Kim B, Takeuchi A, Matsuoka S (2013) Mitochondrial NCX controls directional migration of B lymphocyte. J Physiol Sci 63:S136

[87] Crompton M, Künzi M, Carafoli E (1977). The calcium-induced and sodium-induced effluxes of calcium from heart mitochondria. Evidence for a sodium-calcium carrier. Eur J Biochem.

[88] Wei AC, Liu T, Cortassa S, Winslow RL, O’Rourke B (2011). Mitochondrial Ca^2+^ influx and efflux rates in guinea pig cardiac mitochondria: low and high affinity effects of cyclosporine A. Biochim Biophys Acta.

[89] Liu T, O’Rourke B (2008). Enhancing mitochondrial Ca^2+^ uptake in myocytes from failing hearts restores energy supply and demand matching. Circ Res.

[90] Kohlhaas M, Liu T, Knopp A, Zeller T, Ong MF, Bohm M, O’Rourke B, Maack C (2010). Elevated cytosolic Na^+^ increases mitochondrial formation of reactive oxygen species in failing cardiac myocytes. Circulation.

[91] Bassani RA, Bassani JW, Bers DM (1994). Relaxation in ferret ventricular myocytes: unusual interplay among calcium transport systems. J Physiol.

[92] Claycomb WC, Lanson NAJ, Stallworth BS, Egeland DB, Delcarpio JB, Bahinski A, Izzo NJJ (1998). HL-1 cells: a cardiac muscle cell line that contracts and retains phenotypic characteristics of the adult cardiomyocyte. Proc Natl Acad Sci U S A.

[93] White SM, Constantin PE, Claycomb WC (2004). Cardiac physiology at the cellular level: use of cultured HL-1 cardiomyocytes for studies of cardiac muscle cell structure and function. Am J Physiol Heart Circ Physiol.

[94] Yang Z, Murray KT (2011). Ionic mechanisms of pacemaker activity in spontaneously contracting atrial HL-1 cells. J Cardiovasc Pharmacol.

[95] Adachi T, Shibata S, Okamoto Y, Sato S, Fujisawa S, Ohba T, Ono K (2013). The mechanism of increased postnatal heart rate and sinoatrial node pacemaker activity in mice. J Physiol Sci.

[96] Palmer AE, Jin C, Reed JC, Tsien RY (2004). Bcl-2-mediated alterations in endoplasmic reticulum Ca^2+^ analyzed with an improved genetically encoded fluorescent sensor. Proc Natl Acad Sci U S A.

[97] Opuni K, Reeves JP (2000). Feedback inhibition of sodium/calcium exchange by mitochondrial calcium accumulation. J Biol Chem.

[98] Khatami M, Houshmand M, Sadeghizadeh M, Eftekharzadeh M, Heidari MM, Saber S, Banihashemi K, Scheiber-Mojdehkar B (2010). Accumulation of mitochondrial genome variations in Persian LQTS patients: a possible risk factor?. Cardiovasc Pathol.

[99] Koopman WJ, Willems PH, Smeitink JA (2012). Monogenic mitochondrial disorders. N Engl J Med.

[100] Yaniv Y, Spurgeon HA, Lyashkov AE, Yang D, Ziman BD, Maltsev VA, Lakatta EG (2012). Crosstalk between mitochondrial and sarcoplasmic reticulum Ca^2+^ cycling modulates cardiac pacemaker cell automaticity. PLoS ONE.

[101] Maltsev VA, Lakatta EG (2009). Synergism of coupled subsarcolemmal Ca^2+^ clocks and sarcolemmal voltage clocks confers robust and flexible pacemaker function in a novel pacemaker cell model. Am J Physiol Heart Circ Physiol.

[102] Himeno Y, Sarai N, Matsuoka S, Noma A (2008). Ionic mechanisms underlying the positive chronotropy induced by beta1-adrenergic stimulation in guinea pig sinoatrial node cells: a simulation study. J Physiol Sci.

[103] Himeno Y, Toyoda F, Satoh H, Amano A, Cha CY, Matsuura H, Noma A (2011). Minor contribution of cytosolic Ca^2+^ transients to the pacemaker rhythm in guinea pig sinoatrial node cells. Am J Physiol Heart Circ Physiol.

[104] Severi S, Fantini M, Charawi LA, DiFrancesco D (2012). An updated computational model of rabbit sinoatrial action potential to investigate the mechanisms of heart rate modulation. J Physiol.

[105] Takeuchi A, Matsuoka S (2014). Mitochondrial Na–Ca exchanger NCLX-mediated mitochondria-sarcoplasmic reticulum Ca crosstalk and cardiomyocyte automaticity. J Physiol Sci.

[106] Guo J, Mitsuiye T, Noma A (1997). The sustained inward current in sino-atrial node cells of guinea-pig heart. Pflügers Arch.

[107] Jurkowitz MS, Brierley GP (1982). H^+^-dependent efflux of Ca^2+^ from heart mitochondria. J Bioenerg Biomembr.

[108] Gunter KK, Zuscik MJ, Gunter TE (1991). The Na^+^-independent Ca^2+^ efflux mechanism of liver mitochondria is not a passive Ca^2+^/2H^+^ exchanger. J Biol Chem.

[109] Jiang D, Zhao L, Clish CB, Clapham DE (2013). Letm1, the mitochondrial Ca^2+^/H^+^ antiporter, is essential for normal glucose metabolism and alters brain function in Wolf-Hirschhorn syndrome. Proc Natl Acad Sci U S A.

[110] Bergemann AD, Cole F, Hirschhorn K (2005). The etiology of Wolf-Hirschhorn syndrome. Trends Genet.

[111] Nowikovsky K, Pozzan T, Rizzuto R, Scorrano L, Bernardi P (2012). Perspectives on: SGP symposium on mitochondrial physiology and medicine: the pathophysiology of LETM1. J Gen Physiol.

[112] Tsai MF, Jiang D, Zhao L, Clapham D, Miller C (2014). Functional reconstitution of the mitochondrial Ca^2+^/H^+^ antiporter Letm1. J Gen Physiol.

